# Seewis hantavirus in common shrew (*Sorex araneus*) in Sweden

**DOI:** 10.1186/s12985-020-01477-w

**Published:** 2020-12-29

**Authors:** Olivia Wesula Lwande, Nahla Mohamed, Göran Bucht, Clas Ahlm, Gert Olsson, Magnus Evander

**Affiliations:** 1 Department of Clinical Microbiology, Virology, Umeå University, Umeå, Sweden; 2 Department of Clinical Microbiology, Infection and Immunology, Umeå University, Umeå, Sweden; 3 Department of Wildlife, Fish, and Environmental Studies, Swedish University of Agricultural Sciences, Umeå, Sweden

**Keywords:** Hantavirus, Shrew, Seewis virus, Cytochrome C oxidase I gene, Cytochrome B gene, Molecular barcoding

## Abstract

**Background:**

Rodent borne hantaviruses are emerging viruses infecting humans through inhalation. They cause hemorrhagic fever with renal syndrome and hemorrhagic cardiopulmonary syndrome. Recently, hantaviruses have been detected in other small mammals such as Soricomorpha (shrews, moles) and Chiroptera (bats), suggested as reservoirs for potential pandemic viruses and to play a role in the evolution of hantaviruses. It is important to study the global virome in different reservoirs, therefore our aim was to investigate whether shrews in Sweden carried any hantaviruses. Moreover, to accurately determine the host species, we developed a molecular method for identification of shrews.

**Method:**

Shrews (n = 198), caught during 1998 in Sweden, were screened with a pan-hantavirus PCR using primers from a conserved region of the large genome segment. In addition to morphological typing of shrews, we developed a molecular based typing method using sequencing of the mitochondrial cytochrome C oxidase I (*COI*) and cytochrome B (*CytB*) genes. PCR amplified hantavirus and shrew fragments were sequenced and phylogenetically analysed.

**Results:**

Hantavirus RNA was detected in three shrews. Sequencing identified the virus as Seewis hantavirus (SWSV), most closely related to previous isolates from Finland and Russia. All three SWSV sequences were retrieved from common shrews (*Sorex araneus*) sampled in Västerbotten County, Sweden. The genetic assay for shrew identification was able to identify native Swedish shrew species, and the genetic typing of the Swedish common shrews revealed that they were most similar to common shrews from Russia.

**Conclusion:**

We detected SWSV RNA in Swedish common shrew samples and developed a genetic assay for shrew identification based on the *COI* and *CytB* genes. This was the first report of presence of hantavirus in Swedish shrews.

## Background

Hantaviruses (family *Hantaviridae*, genus *Orthohantavirus*) are found in many different rodents, but also in Soricomorpha (shrews, moles) and Chiroptera (bats) [1]. The rodent borne hantaviruses can infect humans via aerosols from rodent secretions and cause severe disease such as hemorrhagic fever with renal syndrome (HFRS) and hemorrhagic cardiopulmonary syndrome (HCPS) with up to 40% case-fatality rate in the latter syndrome [2]. The relatively recent discovery of the diversity of hantaviruses and their hosts have shed light on the evolutionary history of hantaviruses. Because hantaviruses infect a diverse range of mammalian hosts, e.g. rodents, shrews, moles and bats, cross-species transmission at multiple scales has probably played an important role in hantavirus evolution [1, 3]. Hantaviruses from soricomorphs and bats has so far not been associated with disease, but it could not be ruled out that some of these have an impact on health.

Puumala virus (PUUV) is so far the only recognized endemic hantavirus in Sweden. PUUV is carried by the bank vole (*Myodes glareolus*) and causes HFRS in central and northern Europe [4–6]. There have only been a few reports of possible presence of other hantaviruses in Sweden. One describes the detection of Seoul virus antibodies and RNA in a pet rat in Sweden, however it is not certain that it was infected in Sweden [7]. Another report suggested presence of Tula virus antibodies in samples from yellow-necked mouse (*Apodemus flavicollis*), but no virus RNA or antigen was detected [8]. In neighboring Finland where PUUV also is endemic, Seewis hantavirus (SWSV) has been found in the common shrew (*Sorex araneus*), Boginia hantavirus in water shrew (*Neomys fodiens*), Asikkala hantavirus in pygmy shrew (*Sorex minitus*), and an Altai-like hantavirus in common shrew [9]. Hantaviruses have been detected and described from shrews in all continents except Australia and Oceania, mostly with the use of pan-hantavirus primers and RT-PCR. The hantaviruses are enveloped, negative strand RNA viruses with three segments, L, M and S, that encode the RNA dependent RNA polymerase (L), glycoproteins (M), and the nucleocapsid protein (S). The L-segment is the most conserved segment and therefore suitable for design of primers for a pan-hantavirus PCR. So far, there is no knowledge whether Swedish shrews are infected by hantaviruses. There are six recognized shrew species in Sweden that potentially could be infected: Common shrew (*Sorex araneus*), Eurasian pygmy shrew (*Sorex minutus*), Eurasian water shrew (*Neomys fodiens*), Laxmann’s shrew (*Sorex caecutiens*), Eurasian least shrew/lesser pygmy shrew (*Sorex minutissimus*), and Taiga shrew (*Sorex isodon*). To better understand the diversity of hantaviruses in soricomorphs in Sweden, we investigated the presence of hantaviruses in Swedish shrews and determined the pathogen and the host species by genetic methods. Hantavirus RNA was detected in shrew samples by using a pan-hantavirus RT-PCR [10]. For accurate identification of shrews, we developed a genetic assay based on morphologically determined species using the cytochrome C oxidase I (*COI*) and cytochrome B (*CytB*) genes.

## Methods

### Shrew sampling

The shrews were archival samples from a sampling carried out in the fall of 1998 in Västerbotten County (64.85°N 17.9°E), Sweden, and the sampling was performed as previously described [11]. All shrews had morphologically been determined to be *Sorex spp*, but no further species specific morphological determination had been performed.

### Hantavirus detection and sequencing

Hantavirus RNA was detected in shrew samples by using a pan-hantavirus RT-PCR as described previously [10]. Briefly, total RNA from shrew lung tissue was prepared by using a Viral NA Extraction Kit (DiaSorin, Dublin, Ireland), according to the manual, with 250 μL of the homogenate and 10 μL of protein kinase K (Qiagen, Hilden, Germany) in 1.5 mL sterile micro-centrifuge tubes. Viral nucleic acid was extracted and collected in an elution volume of 50 μL. Obtained RNA was used for RT-PCR with pan-hantavirus primers as previously described [10]. The RT-PCR products were sequenced (GATC sequencing company, Germany) and the obtained sequences were compared with available sequences in GenBank to determine the identity of the hantavirus. Phylogenetic analysis was performed using Molecular Evolutionary Genetic Analysis (MEGA6).

### Shrew genetic typing

Shrews for genetic typing were acquired from already morphologically species determined shrews. Genetic typing of shrews was performed targeting the barcoding regions of the *COI* and *CytB* genes, using:

- sorcoi29F 5′-CCGGAATAGTAGGVACAGCCCT-3′ and
- sorcoi380R 5′-ACAGATGCTCCTGCRTGGGC-3′;
- sorcytb365F 5′ CAGTAATAGCCACTGCCTTTATAGG-3′ and
- sorcytb969R 5′-CATTGGCTGAATGGGCGGAATATTAT-3′ primers respectively.

The PCR amplification was performed by using the KAPA Taq PCR kit (KAPA Biosystems, Boston, MA, USA). The optimized cycling conditions after an initial denaturation at 95 °C for 3 min were; 35 cycles at 95 °C for 3 s, annealing at 55 °C for 30 s and extension at 72 °C for 1 min, before standby at 4 °C. The PCR products were visualized on 1.2% agarose gels (containing GelRed; Biotium, Inc.), purified by NucleoSpin Gel, PCR Clean-up kit (Macherey–Nagel, Düren, Germany) and sequenced (GATC sequencing company, Germany). The obtained sequences were compared with available sequences in GenBank to determine the identity of the shrew species. Phylogenetic analysis was performed using MEGA6 [12] and the Maximum Likelihood method based on the Tamura-Nei model [13].

## Results

### Hantavirus detection in shrews

**Fig. 1:**
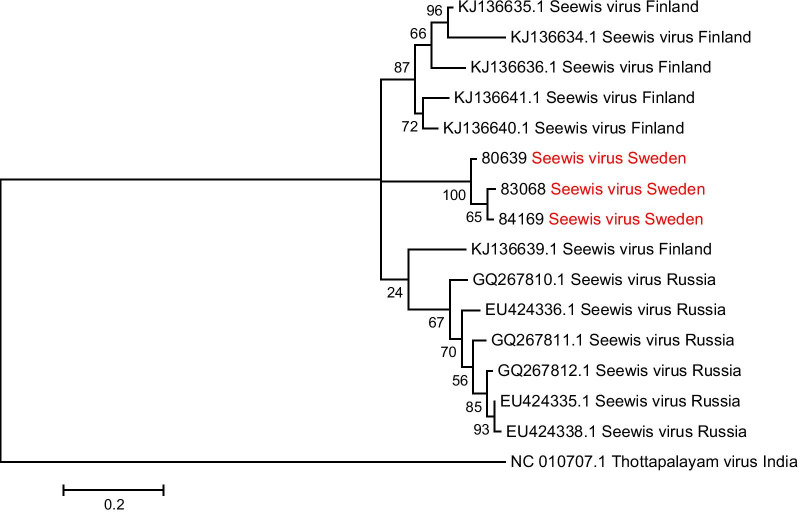
Phylogenetic relationship between the RNA sequences of Seewis virus (SWSV) detected sequences in the study (80639, 83068, 84169), based on the partial L-segment sequence. The isolates obtained from Swedish shrews are marked in red. The tree was generated by MEGA6 [12] using the Maximum Likelihood method based on the Tamura-Nei model [13]. The values on the branches indicate bootstrap values. Initial tree(s) for the heuristic search were obtained by applying the Neighbor-Joining method to a matrix of pairwise distances estimated using the Maximum Composite Likelihood (MCL) approach. The tree is drawn to scale, with branch lengths measured in the number of substitutions per site. The analysis involved 16 nucleotide sequences. Codon positions included were 1st + 2nd + 3rd + Noncoding. There was a total of 273 positions in the final dataset

Pan-hantavirus primers were used to screen lung tissue from the 198 shrews. In three shrews, an RT-PCR product with the predicted hantavirus specific size was detected, and sequencing revealed that all three carried SWSV RNA. All SWSV RNA were detected in common shrews (*Sorex araneus*) sampled in Västerbotten County, Sweden. The SWSV sequences from the 459 bp fragment from the L-segment were closely related to each other, and all of them were similar to common shrew L-segment sequences originating from Finland and Russia (Fig. 1).

### Barcode-based sequence identification of shrews

**Fig. 2:**
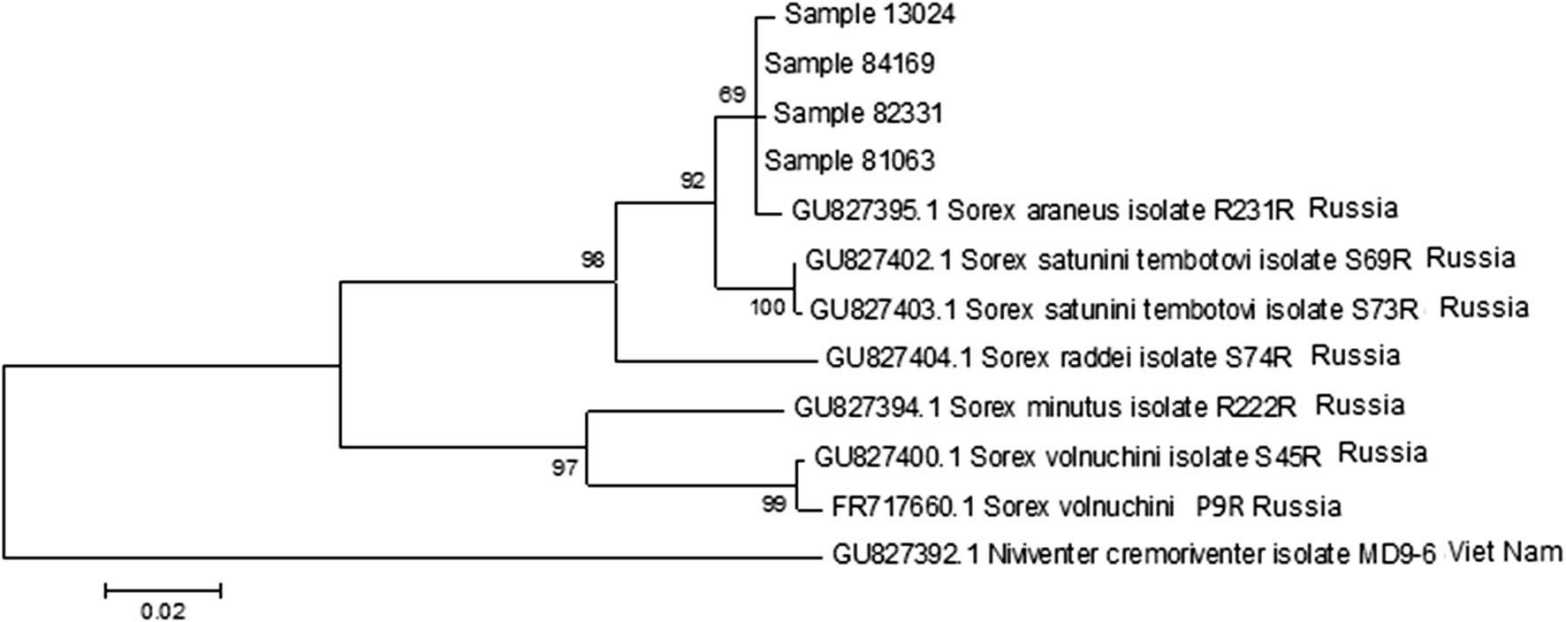
Phylogenetic relationship between the DNA sequences of study shrews (13024, 84169, 82331, 81063) and previously sequenced shrews based on cytochrome b (*CytB*) gene. The tree was generated by MEGA6 [12] using the using the Maximum Likelihood method based on the Tamura-Nei model [13]. The values on the branches indicate bootstrap values. The percentage of trees in which the associated taxa clustered together is shown next to the branches. Initial tree(s) for the heuristic search were obtained by applying the Neighbor-Joining method to a matrix of pairwise distances estimated using the Maximum Composite Likelihood (MCL) approach. The tree was drawn to scale, with branch lengths measured in the number of substitutions per site. The analysis involved 12 nucleotide sequences. Codon positions included were 1st + 2nd + 3rd + Noncoding. There was a total of 604 positions in the final dataset

**Fig. 3:**
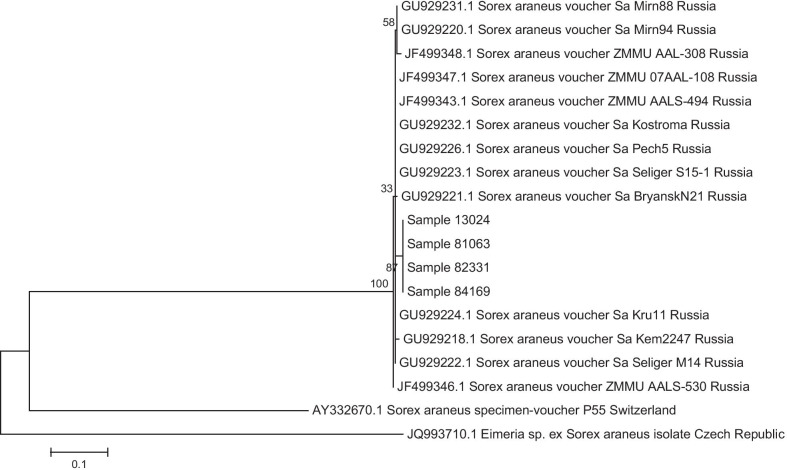
Phylogenetic relationship between the DNA sequences of study shrews (13024, 84169, 82331, 81063) and previously sequenced shrews based on cytochrome oxidase I (*COI*) gene. The tree was generated by MEGA6 [12] using the using the Maximum Likelihood method based on the Tamura 3-parameter model [13]. The values on the branches indicate bootstrap values. The percentage of trees in which the associated taxa clustered together is shown next to the branches. Initial tree(s) for the heuristic search were obtained automatically by applying the Maximum Parsimony method. The tree is drawn to scale, with branch lengths measured in the number of substitutions per site. The analysis involved 19 nucleotide sequences. Codon positions included were 1st + 2nd + 3rd + Noncoding. There was a total of 393 positions in the final dataset

Five shrew species (*Sorex araneus, Sorex minutus, Sorex caecutiens, Neomys fodiens, Sorex minutissimus*) of the six known species in Sweden were used for the development of the DNA barcoding assay. The shrews had previously been morphologically species determined. After amplification with the *COI* and *CytB* shrew-primers, the PCR products were sequenced. The sequence correlated with both the morphologically determined species, and previously published sequences for all five shrew species. Thus, genetic typing of shrews in Sweden was accomplished. Using a similar barcoding approach as for the five shrew species, amplifications of both *CytB* (604 bp) and *COI* (393 bp) gene fragments were performed for one of the SWSV positive shrews and three other shrews from the sampling. Unfortunately, there was not enough material left to perform a thorough analysis of all samples. The sequencing revealed that all four shrews from the sampling in Västerbotten County were *Sorex araneus,* and phylogeny analysis revealed closest relation to *Sorex araneus* isolates from Russia (Figs. 2, 3).

## Discussion

In here, we detected SWSV RNA in Swedish shrew samples and developed a genetic assay for shrew identification based the *COI* and *CytB* genes. This was the first report of presence of hantavirus in Swedish shrews.

So far, SWSV has been detected in several European countries, and also in Siberian part of Russia. The common shrew, *Sorex araneus,* as well as SWSV is widespread, extending from Central Siberia to Western Europe. Shrews have been collected in Sweden in different projects, but, as far as we know, have not been screened for hantaviruses. Based on the available partial sequences detected in the archival shrew samples, the Swedish SWSV sequences from the shrews sampled 1998, were most closely related to Russian and Finnish SWSV, and the three SWSV sequence from Sweden were all in the same cluster. The low supportive analysis just based on the partial L-segment is inadequate, and more full-genome sequences are needed to acquire a more thorough understanding of the phylogenetic relationships between SWSV in different geographic regions. Full genome sequences were however difficult to obtain from the archival tissue samples. Hantaviruses are also notoriously difficult to isolate in cell culture, and thus, it is cumbersome to sequence the full genomes. Due to the low sample numbers from other shrews than the common shrew, we cannot exclude that SWSV and other hantaviruses could be found also from these other species in Sweden. The sequences generated from common shrews that were analysed in the study, were most closely related to Russian shrew sequences. Genetic typing of shrews is important for species determination in archival samples, and in projects where morphological typing is not available. In conclusion, the study have identified SWSV hantavirus in common shrews in Sweden, thus adding this geographical region to the prevalence of SWSV, as well as showing the close relation with other SWSV in Russia and Finland.

## Conclusions

We could show presence of SWSV RNA in Swedish common shrew samples, and the Swedish SWSV were most related to Finnish and Russian SWSW. Furthermore, we developed a genetic assay for shrew identification based on the *COI* and *CytB* genes. As far as we know, this was the first report of presence of hantavirus in Swedish shrews, and increases the understanding of the diversity of hantaviruses and their hosts.

## Data Availability

The datasets supporting the conclusions regarding sequences in this article will be available in GenBank (https://www.ncbi.nlm.nih.gov/genbank/) with accession numbers MT939451-61. The remaining datasets used and/or analysed during the current study are available from the corresponding author on reasonable request.

## Abbreviations

*COI*: Cytochrome C oxidase I
*CytB*: Cytochrome B
DNA: Deoxyribonucleic acid
HFRS: Hemorrhagic fever with renal syndrome
HCPS: Hemorrhagic cardiopulmonary syndrome
MEGA6: Molecular evolutionary genetic analysis
PCR: Polymerase chain reaction
PUUV: Puumala virus
RNA: Ribonucleic acid
RT-PCR: Reverse-transcriptase polymerase chain reaction
SWVS: Seewis virus

## References

[1] Holmes EC, Zhang YZ (2015). The evolution and emergence of hantaviruses. Curr Opin Virol.

[2] Jonsson CB, Figueiredo LT, Vapalahti O (2010). A global perspective on hantavirus ecology, epidemiology, and disease. Clin Microbiol Rev.

[3] Arai S, Yanagihara R (2020). Genetic diversity and geographic distribution of bat-borne hantaviruses. Curr Issues Mol Biol.

[4] Pettersson L, Boman J, Juto P, Evander M, Ahlm C (2008). Outbreak of Puumala virus infection. Sweden Emerg Infect Dis.

[5] Olsson GE, Dalerum F, Hörnfeldt B, Elgh F, Palo TR, Juto P (2003). Human hantavirus infections, Sweden. Emerg Infect Dis.

[6] Vapalahti O, Mustonen J, Lundkvist A, Henttonen H, Plyusnin A, Vaheri A (2003). Hantavirus infections in Europe. Lancet Infect Dis.

[7] Lundkvist A, Verner-Carlsson J, Plyusnina A, Forslund L, Feinstein R, Plyusnin A (2013). Pet rat harbouring Seoul hantavirus in Sweden, June 2013. Euro Surveill.

[8] Lõhmus M, Verner-Carlsson J, Borg O, Albihn A, Lundkvist Å (2016). Hantavirus in new geographic regions, Sweden. Infect Ecol Epidemiol.

[9] Ling J, Sironen T, Voutilainen L, Hepojoki S, Niemimaa J, Isoviita VM (2014). Hantaviruses in Finnish soricomorphs: evidence for two distinct hantaviruses carried by *Sorex araneus* suggesting ancient host-switch. Infect Genet Evol.

[10] Mohamed N, Nilsson E, Johansson P, Klingström J, Evander M, Ahlm C (2013). Development and evaluation of a broad reacting SYBR-green based quantitative real-time PCR for the detection of different hantaviruses. J Clin Virol.

[11] Olsson GE, White N, Hjältén J, Ahlm C (2005). Habitat factors associated with bank voles (*Clethrionomys glareolus*) and concomitant hantavirus in northern Sweden. Vector Borne Zoonotic Dis.

[12] Tamura K, Stecher G, Peterson D, Filipski A, Kumar S (2013). MEGA6: molecular evolutionary genetics analysis version 6.0. Mol Biol Evol.

[13] Tamura K, Nei M (1993). Estimation of the number of nucleotide substitutions in the control region of mitochondrial DNA in humans and chimpanzees. Mol Biol Evol.

